# Supplementary material for: The Small RNA Universe of Capitella teleta
Source: Front Mol Biosci. 2022 Feb 25;9:802814. doi: 10.3389/fmolb.2022.802814 (PMC8915122; doi:10.3389/fmolb.2022.802814)
Supplement: Supplementary file 1 [file DataSheet1.ZIP › Supplement/known/CAPTEscaffold_777_28939.pdf]

| | | |
|---|---|---|
| Provisional ID | : | CAPTEscaffold_777_28939 |
| Score total | : | 6776.7 |
| Score for star read(s) | : | 3.9 |
| Score for read counts | : | 6766.5 |
| Score for mfe | : | 1.7 |
| Score for randfold | : | 1.6 |
| Score for cons. seed | : | 3 |
| Total read count | : | 13284 |
| Mature read count | : | 10850 |
| Loop read count | : | 0 |
| Star read count | : | 2434 |

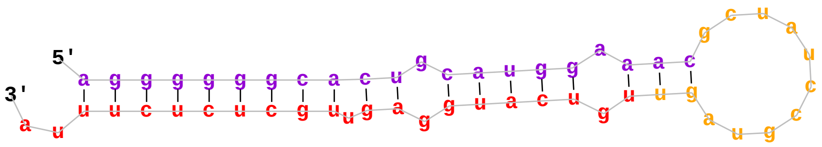


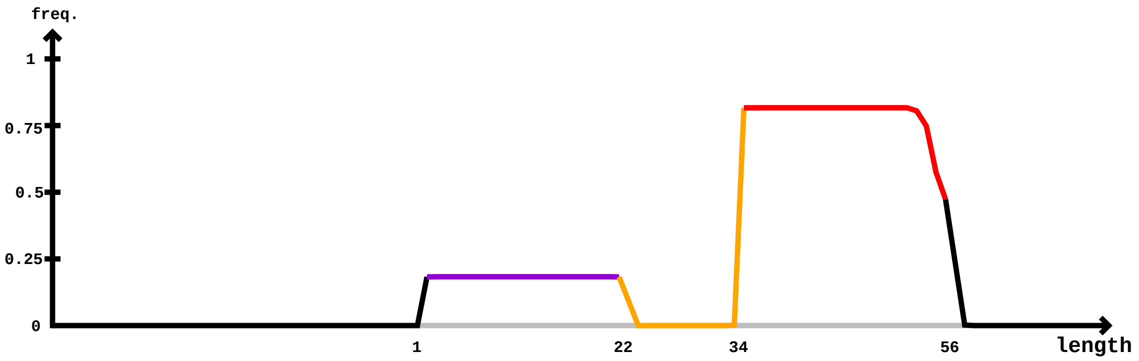


Star Mature

```
5'- gcccuucugacugcaggauucaaucggcuggucguucaggggggcacugcauggaaacgcuauccguaguugucauggaguugcucucuuuaacgaccugaccuaauu -3' obs
    gcccuucugacugcaggauucaaucggcuggucguucaggggggcacugcauggaaacgcuauccguaguugucauggaguugcucucuuuaacgaccugaccuaauu     exp
    gcccuucugacugcaggauucaaucggcuggucguucaggggggcacugcauggaaacgcuauccguaguugucauggaguugcucucuuuaacgaccugaccuaauu     known
    ....(((((....))))).......(((.(((((((.(((((((((((.(((((.(((..........))).))))).)).))))))))).))))))).).)).....  reads  mm  sample
    .....................................aggggggcacuAcauggaaa...................................................  1      1   seq
    .....................................aggggggcacugcauggaaa...................................................  8      0   seq
    .....................................agggUggcacugcauggaaac..................................................  1      1   seq
    .....................................Ngggggggcacugcauggaaac.................................................  2      1   seq
    .....................................aggggAgcacugcauggaaac..................................................  2      1   seq
    .....................................aggggggcacugcauggaaaA..................................................  1      1   seq
    .....................................aggggggcacCgcauggaaac..................................................  1      1   seq
    .....................................aggggggcacugcaugAaaac..................................................  1      1   seq
    .....................................aggggNgcacugcauggaaac..................................................  1      1   seq
    .....................................agAggggcacugcauggaaac..................................................  4      1   seq
    .....................................aggggggcacugcGuggaaac..................................................  2      1   seq
    .....................................agggAggcacugcauggaaac..................................................  3      1   seq
    .....................................aggggggcacugcauAgaaac..................................................  2      1   seq
    .....................................aggggggcCcugcauggaaac..................................................  1      1   seq
    .....................................aAgggggcacugcauggaaac..................................................  2      1   seq
    .....................................aggggggAacugcauggaaac..................................................  1      1   seq
    .....................................aggUgggcacugcauggaaac..................................................  1      1   seq
    .....................................Gggggggcacugcauggaaac..................................................  30     1   seq
    .....................................aggggggcGcugcauggaaac..................................................  2      1   seq
    .....................................aggAgggcacugcauggaaac..................................................  3      1   seq
    .....................................aUggggggcacugcauggaaac.................................................  1      1   seq
    .....................................aggggggcacugAauggaaac..................................................  3      1   seq
    .....................................aggggggcUcugcauggaaac..................................................  1      1   seq
    .....................................aggggggcacugcauggCaac..................................................  1      1   seq
    .....................................aggggggUacugcauggaaac..................................................  1      1   seq
    .....................................aggggggcacugcauggaaac..................................................  2272   0   seq
    .....................................aggggggcacugcaAggaaac..................................................  1      1   seq
    .....................................aggggggcacugcauggaaacA.................................................  77     1   seq
    ......................................Agggggcacugcauggaaac..................................................  6      1   seq
    .......................................Aggggcacugcauggaaac..................................................  2      1   seq
    .....................................................................uugucauggaguugcucucu...................  3      0   seq
    .....................................................................uugucauggaguugcucucuu..................  9      0   seq
```

Star Mature

```
gcccuucugacugcaggauucaaucggcuggucguucagggggcacugcauggaaacgcuauccguaguugucauggaguugcucucuuuaacgaccugaccuaauu
....................................................................uugucauggaguugcucucuuu.................	1	0	seq
....................................................................uugucauggaguugcucucuuua................	8	0	seq
....................................................................Cugucauggaguugcucucuuuaa...............	1	1	seq
.....................................................................ugucauggaguugcucuU....................	1	1	seq
.....................................................................ugucauAgaguugcucuc....................	1	1	seq
.....................................................................ugucaGggaguugcucuc....................	1	1	seq
.....................................................................ugucauggaguugcucuA....................	1	1	seq
.....................................................................Ggucauggaguugcucuc....................	1	1	seq
.....................................................................ugucauggaguugcucuc....................	151	0	seq
.....................................................................Ggucauggaguugcucucu...................	1	1	seq
.....................................................................ugucauggaguugcucucu...................	731	0	seq
.....................................................................ugucauggaguugcuGucu...................	1	1	seq
.....................................................................ugAcauggaguugcucucu...................	3	1	seq
.....................................................................Agucauggaguugcucucu...................	4	1	seq
.....................................................................ugucauggGguugcucucu...................	1	1	seq
.....................................................................ugucauggaguugcucuUu...................	1	1	seq
.....................................................................ugucauggaguugcucucA...................	1	1	seq
.....................................................................ugucauggaguugcucAcu...................	1	1	seq
.....................................................................ugucauAgaguugcucucu...................	2	1	seq
.....................................................................ugucauggagAugcucucu...................	1	1	seq
.....................................................................uguUauggaguugcucucu...................	1	1	seq
.....................................................................ugucauggaguugcucucAu..................	1	1	seq
.....................................................................ugucauggaguugcucucuu..................	2258	0	seq
.....................................................................ugucGuggaguugcucucuu..................	1	1	seq
.....................................................................ugucauAgaguugcucucuu..................	6	1	seq
.....................................................................ugAcauggaguugcucucuu..................	5	1	seq
.....................................................................ugucauggGguugcucucuu..................	1	1	seq
.....................................................................ugucauggaguugcuAucuu..................	1	1	seq
.....................................................................ugucauggaguugAucucuu..................	2	1	seq
.....................................................................Ggucauggaguugcucucuu..................	2	1	seq
.....................................................................ugucaAggaguugcucucuu..................	1	1	seq
.....................................................................Agucauggaguugcucucuu..................	5	1	seq
.....................................................................ugucauggaguuUcucucuuu.................	1	1	seq
.....................................................................ugucauggaguugcucucuuA.................	2	1	seq
.....................................................................ugucauggagAugcucucuuu.................	2	1	seq
.....................................................................ugucauggaguugcucucuuu.................	1333	0	seq
.....................................................................ugucauggGguugcucucuuu.................	1	1	seq
.....................................................................ugucauAgaguugcucucuuu.................	2	1	seq
.....................................................................ugucauUgaguugcucucuuu.................	1	1	seq
.....................................................................Agucauggaguugcucucuuu.................	6	1	seq
.....................................................................ugucauggaguugcucAcuuu.................	2	1	seq
.....................................................................ugucauggaguAgcucucuuu.................	1	1	seq
.....................................................................ugAcauggaguugcucucuuu.................	7	1	seq
.....................................................................ugucauggagGugcucucuuua................	1	1	seq
.....................................................................ugucauggaguugcCucuuua................	1	1	seq
.....................................................................ugucauggaguugcucuUuuua................	1	1	seq
.....................................................................ugucauggaguCgcucucuuua................	1	1	seq
.....................................................................ugucauggaguAgcucucuuua................	3	1	seq
.....................................................................ugucauggCguugcucucuuua................	1	1	seq
.....................................................................ugucauggaguugcucucuGua................	2	1	seq
.....................................................................ugucaCggaguugcucucuuua................	1	1	seq
.....................................................................ugucauggaguugcucucuuua................	4395	0	seq
.....................................................................ugucauUgaguugcucucuuua................	1	1	seq
.....................................................................ugAcauggaguugcucucuuua................	11	1	seq
.....................................................................ugucauggaguuCcucucuuua................	1	1	seq
.....................................................................ugucauAgaguugcucucuuua................	12	1	seq
.....................................................................ugucauggaguugcucucuuCa................	2	1	seq
.....................................................................ugucauggaguugcucucuuAa................	3	1	seq
.....................................................................ugucauggaguugcucAcuuua................	3	1	seq
.....................................................................ugucauggaguugcucucuuuU................	22	1	seq
.....................................................................ugucaAggaguugcucucuuua................	4	1	seq
.....................................................................ugucauggaguugcucucuCua................	1	1	seq
.....................................................................ugucauggaguuUcucucuuua................	2	1	seq
.....................................................................ugucGuggaguugcucucuuua................	1	1	seq
.....................................................................Ngucauggaguugcucucuuua................	1	1	seq
.....................................................................uAucauggaguugcucucuuua................	1	1	seq
.....................................................................uguUauggaguugcucucuuua................	1	1	seq
.....................................................................ugucauggaguugcucucuuuG................	1	1	seq
.....................................................................ugucauggaguugUucucuuua................	1	1	seq
.....................................................................Ggucauggaguugcucucuuua................	2	1	seq
```

Star Mature

```
gcccuucugacugcaggauucaaucggcuggucguucaggggggcacugcauggaaacgcuauccguaguugucauggaguugcucucuuuaacgaccugaccuaauu
......................................................................ugucaugCaguugcucucuuua................   1    1    seq
......................................................................uCucauggaguugcucucuuua................   1    1    seq
......................................................................ugCcauggaguugcucucuuua................   1    1    seq
......................................................................ugucauggaguugAucucuuua................   1    1    seq
......................................................................ugucauggaguuAcucucuuua................   1    1    seq
......................................................................ugucaugAaguugcucucuuua................   1    1    seq
......................................................................ugucauggGguugcucucuuua................   1    1    seq
......................................................................Agucauggaguugcucucuuua................   13   1    seq
......................................................................ugucauggaguugcucuUuuuaa...............   1    1    seq
......................................................................Ggucauggaguugcucucuuuaa...............   1    1    seq
......................................................................uCucauggaguugcucucuuuaa...............   1    1    seq
......................................................................ugucauggaguugcucucuuuaG...............   1    1    seq
......................................................................ugucauggaguugcucucuuuaN...............   1    1    seq
......................................................................ugucauCgaguugcucucuuuaa...............   1    1    seq
......................................................................ugucauggUguugcucucuuuaa...............   1    1    seq
......................................................................ugucauggaguugcucucuuAaa...............   1    1    seq
......................................................................ugucauggaguugcucucuuuaU...............   895  1    seq
......................................................................Agucauggaguugcucucuuuaa...............   3    1    seq
......................................................................ugucauggaguugcucucuuuaC...............   2    1    seq
......................................................................ugucauggaguugcucucuuuaa...............   853  0    seq
......................................................................ugucauAgaguugcucucuuuaa...............   1    1    seq
......................................................................ugucauggaguugcucucuuuaaU..............   1    1    seq
......................................................................ugucauggaguugcucucuuuaaA..............   23   1    seq
........................................................................ucauggaguugcucucuuua................   1    0    seq
```
